# Workplace Violence Safety Perception, Occupational Stress, and Emotional Exhaustion Among Nurses: Relationships with Psychological Resilience

**DOI:** 10.3390/healthcare14213826

**Published:** 2026-11-09

**Authors:** Ahmet Aktar, Süreyya Yiğitalp Rençber

**Affiliations:** 1 Batman Training and Research Hospital, Batman 72070, Türkiye; 2 Department of Health Management, Faculty of Health Sciences, Batman University, Batman 72060, Türkiye

**Keywords:** workplace violence, psychological resilience, occupational stress, emotional exhaustion, nurses, occupational health

## Abstract

**Background:** Nurses’ perceptions of trust and safety regarding violence in healthcare may be related to occupational stress and emotional exhaustion. This study aimed to examine the relationships between workplace violence safety perception, occupational stress, emotional exhaustion, and psychological resilience among nurses. **Methods:** This cross-sectional and correlational study was conducted with 419 nurses working in public hospitals. The questionnaire included a sociodemographic form and four standardized scales. Pearson correlation and regression-based indirect-effect analyses were performed. **Results:** Occupational stress was positively associated with emotional exhaustion, whereas psychological resilience was negatively associated with both occupational stress and emotional exhaustion. Indirect-effect analyses showed statistically significant indirect associations between workplace violence safety perception and both occupational stress and emotional exhaustion when psychological resilience was included in the models. **Conclusions:** The findings suggest that workplace violence safety perception, psychological resilience, occupational stress, and emotional exhaustion are interrelated among nurses. More positive perceptions of workplace violence safety were associated with greater psychological resilience, while higher psychological resilience was associated with lower levels of occupational stress and emotional exhaustion. Because of the cross-sectional design, these findings should be interpreted as associations rather than causal relationships. **Implications for Practice:** Healthcare institutions should develop security policies to prevent violence, effective incident reporting systems, psychosocial support services, and programs that strengthen psychological resilience.

## 1. Introduction

The International Labour Organization defines workplace violence as any act, incident, or behavior that exceeds reasonable limits of conduct, such as an individual being assaulted, threatened, harmed, or injured during or as a direct result of their work [1]. Workplace violence, which is increasing every year in the health sector, has become a global problem today [2,3]. Workplace violence against healthcare workers is encountered throughout their professional lives and is considered a significant occupational health and safety problem at the global level [4]. Nurses, being among the professional groups that have the closest and longest contact with patients in the delivery of healthcare services, are one of the most important targets or victims of workplace violence. Therefore, they are among the healthcare workers most affected by workplace violence. The literature indicates that healthcare workers are at a higher risk of experiencing violence compared to other service sector employees and that nurses and nursing assistants are among the most at-risk groups within the healthcare sector [5,6]. Healthcare workers are sixteen times more likely to experience violence than employees in other service sectors [7]. A systematic review and meta-analysis study indicated that a significant proportion of healthcare workers are exposed to various types of violence, including physical violence, verbal violence, threats, and sexual harassment [8]. Violence in healthcare can arise not only from individual reasons but also from problems related to the functioning of healthcare services. Long waiting times, the expectation of urgency among patients and their relatives, distrust in service delivery, communication problems, the emotional sensitivity of patients’ relatives, inadequate security measures, and problems related to health policies are among the main factors associated with an increased risk of violence [9,10,11]. Violence in healthcare includes not only a physical attack or security problem experienced at the time of the incident, but also negative situations affecting psychological health. Being exposed to violence or working with the constant fear of encountering violence can negatively affect both the physical and psychological health of healthcare workers [12,13]. Previous studies have reported that occupational stress among nurses is associated with burnout, depression, reduced work motivation, and intentions to leave their jobs [14,15,16,17]. The World Health Organization has also identified workplace violence as an important occupational concern for health workers, with implications for their well-being and the delivery of healthcare services [7].

The physical and emotional safety of nurses is fundamental to their ability to cope with workplace violence and create a healthy work environment. High levels of trust among employees ensure they feel safe both physically and emotionally. Strengthening this sense of security may allow employees to focus better on their work and may be associated with lower levels of job dissatisfaction, burnout, and occupational stress. A safe environment makes it easier for healthcare workers to cope with risks like violence, supporting their job performance and overall psychological well-being. In this sense, psychological resilience is seen as an important protective resource. It has been stated that individuals with high psychological resilience can adapt better to such challenging conditions and cope with stress more effectively [18,19,20]. Although previous studies suggest that psychological resilience may serve as a protective factor in stress and burnout processes, studies examining the relationships among nurses’ perceptions of trust and safety regarding violence in healthcare, psychological resilience, occupational stress, and emotional exhaustion remain limited [18,20]. Therefore, psychological resilience may be considered an important psychological resource associated with nurses’ perceptions of workplace safety, occupational stress, and emotional exhaustion. This study was planned based on this gap. The aim of this study was to examine the relationships among workplace violence safety perception, occupational stress, emotional exhaustion, and psychological resilience among nurses working in public hospitals. The findings of this study may contribute to the development of practices and policies aimed at preventing violence in healthcare institutions, strengthening employee safety, and providing psychosocial support for nurses.

### Study Hypotheses

The hypotheses of the study were determined as follows:

**H1.**  *Nurses’ workplace violence safety perception is positively related to their psychological resilience.*

**H2.**  *The level of psychological resilience is negatively related to the level of occupational stress.*

**H3.**  *The level of psychological resilience is negatively related to emotional exhaustion.*

**H4.**  *Workplace violence safety perception is negatively related to occupational stress.*

**H5.**  *Workplace violence safety perception is negatively related to emotional exhaustion.*

**H6.**  *A statistically significant indirect association involving psychological resilience is expected between workplace violence safety perception and occupational stress.*

**H7.**  *A statistically significant indirect association involving psychological resilience is expected between workplace violence safety perception and emotional exhaustion.*

## 2. Materials and Methods

This study was conducted to examine the relationships between workplace violence safety perception, occupational stress, emotional exhaustion, and psychological resilience among nurses working in public hospitals. The study was carried out using a cross-sectional and correlational design based on a quantitative research approach. In the theoretical model of the study, it was expected that workplace violence safety perception, occupational stress, emotional exhaustion, and psychological resilience would be associated with one another. Therefore, psychological resilience was included in the indirect-effect analyses together with workplace violence safety perception, occupational stress, and emotional exhaustion.

### 2.1. Study Design and Sample Size

The study population consisted of nurses working in two public hospitals located in Batman city center, Türkiye. The sample was determined using a convenience sampling method. Nurses who had been working in a public hospital for at least one year and agreed to participate in the study were included. A total of 424 questionnaires were obtained. Five questionnaires were excluded due to incomplete data, leaving 419 complete cases for the final analyses. No formal a priori sample-size calculation was performed, and post-hoc power analysis was therefore not used to justify the adequacy of the final sample.

### 2.2. Data Collection Tools and Variables

The data collection process was conducted using both online and face-to-face methods, taking into account the demanding working conditions of nurses. Data were collected between 25 November 2025 and 31 January 2026, following ethical approval obtained on 13 November 2025 (Decision No. 2025/11-04). Data collection initially began online. As the number of online responses was insufficient, face-to-face data collection was subsequently conducted. To reduce the risk of duplicate responses, nurses approached face-to-face were asked whether they had previously completed the online survey, and those who had were not invited to participate again. Nurses were invited to the study through social media platforms. The survey link was shared along with an informed consent form and a summary of the research objective. During face-to-face data collection, eligible nurses from all clinical units of the participating hospitals were approached at suitable times. The researcher briefly explained the study, obtained informed consent, and administered the questionnaire in person. Overall, 419 valid responses were obtained from nurses working in two public hospitals in Batman city center, Türkiye. Because recruitment involved both online and face-to-face convenience sampling, the exact number of nurses who received or viewed the study invitation could not be determined; therefore, a response rate could not be calculated. The survey form included a Sociodemographic Information Form, the Occupational Stress Scale, the Brief Psychological Resilience Scale, the Work-Related Emotional Exhaustion Scale, and the Health Violence-Related Security-Trust Perception Scale. The sociodemographic information form collected data on participants’ age, gender, education level, marital status, income level, and professional experience.

Occupational stress was assessed using the four-item Perceived Occupational Stress Scale developed by Marcatto et al. [21] and adapted into Turkish by Yıldırım et al. [22]. Items are rated on a five-point Likert scale (1 = strongly disagree, 5 = strongly agree). In the present study, item scores were summed to obtain a total score ranging from 4 to 20, with higher scores indicating greater perceived occupational stress. Cronbach’s alpha was 0.85 in the Turkish adaptation study and 0.855 in the present study.

Psychological resilience was assessed using the six-item Brief Resilience Scale developed by Smith et al. [23] and adapted into Turkish by Doğan [24]. Items are rated on a five-point Likert scale (1 = strongly disagree, 5 = strongly agree). After reverse-scored items are recoded, total scores range from 6 to 30, with higher scores indicating greater psychological resilience. Cronbach’s alpha was 0.83 in the Turkish adaptation study and 0.768 in the present study.

Work-related emotional exhaustion was assessed using the six-item Emotional Exhaustion Scale developed by Wharton [25], based on the Maslach and Jackson [26] burnout framework, and adapted into Turkish by Günay [27]. Items are rated on a five-point Likert scale ranging from 1 (I never feel this way at work) to 5 (I feel this way every day), yielding a total score ranging from 6 to 30. Higher scores indicate greater work-related emotional exhaustion. Cronbach’s alpha was 0.903 in the Turkish adaptation study and 0.931 in the present study.

Workplace violence safety perception was assessed using the seven-item Workplace Violence Safety Perception Scale developed by Kowalenko et al. [28] and adapted into Turkish by Şengül et al. [29]. The scale is rated on a 10-point response format and consists of two subdimensions: Safety (three items) and Violence Management (four items). After reverse scoring the relevant items, the scores are summed to obtain a total score ranging from 7 to 70. Scores range from 3 to 30 for the Safety subdimension and from 4 to 40 for the Violence Management subdimension. Higher scores indicate more positive perceptions of workplace violence safety and trust. The total score was used in the present study because the Turkish validation study also reported and evaluated an overall scale score in addition to the two subdimension scores [29]. In the Turkish adaptation study, Cronbach’s alpha was 0.845 for the total scale, 0.806 for the safety dimension, and 0.839 for the confidence in violence management dimension. In the present study, Cronbach’s alpha for the total scale was 0.742 and McDonald’s omega was 0.767.

### 2.3. Data Analysis

The distributions of occupational stress, psychological resilience, emotional exhaustion, and workplace violence safety perception were first assessed using skewness and kurtosis coefficients together with Q–Q plots. Values within ±2 and an approximately linear pattern in the Q–Q plots were considered to indicate an approximately normal distribution [30].

As part of the preliminary analyses, the internal consistency of the measurement instruments was assessed using Cronbach’s alpha (α) and McDonald’s omega (ω) coefficients. For the Workplace Violence Safety Perception Scale, internal consistency coefficients were also calculated separately for the safety and violence management subdimensions. Five questionnaires with incomplete data were excluded, leaving 419 complete cases for the analyses.

Group comparisons were conducted by sex and shift-work duration using independent-samples *t*-tests and one-way ANOVA, respectively. Effect sizes were calculated using Hedges’ *g* for independent-samples *t*-tests and omega-squared (ω^2^) for one-way ANOVA. Hedges’ *g* values of 0.20, 0.50, and 0.80 were interpreted as small, medium, and large effects, respectively, whereas ω^2^ values of 0.01, 0.06, and 0.14 were interpreted as small, medium, and large effects, respectively [31,32].

Descriptive statistics included the mean, standard deviation, median, minimum, and maximum values. Pearson correlation analysis was used to examine linear relationships among the study variables, and the results were visualized using a correlation matrix. The visualization was performed using R [33]. The *psych* package [34] was used to calculate correlation coefficients and significance levels, while the *ggcorrplot* package [35] was used to visualize the correlation matrix.

Indirect associations involving psychological resilience in the relationships between workplace violence safety perception and occupational stress and emotional exhaustion were examined using Hayes’ regression-based approach [36]. The analyses were conducted using PROCESS Macro v4.2, Model 4. Indirect effects were estimated using 5000 bootstrap samples with 95% confidence intervals. The models were adjusted for sex, age group, work experience, and shift duration. Both unstandardized (B) and standardized (β) coefficients were reported. Workplace violence safety perception was specified as the independent variable (X), psychological resilience as the intervening variable (M), and occupational stress and emotional exhaustion as outcome variables (Y) in separate models. Unstandardized coefficients (B), standardized coefficients (β), and direct, total, and indirect associations were reported. An indirect association was considered statistically significant when the bootstrap confidence interval did not include zero [36].

The assumptions underlying the regression-based indirect-association models were examined before the analyses. Linearity, normality of residuals, homoscedasticity, independence of errors, multicollinearity, and influential observations were assessed. Standardized residuals approximately followed the diagonal line in the normal P–P plots, and no clear systematic pattern was observed in scatterplots of standardized residuals against predicted values. Tolerance values ranged from 0.477 to 0.962, and variance inflation factor (VIF) values ranged from 1.039 to 2.096, indicating no problematic multicollinearity. Durbin–Watson statistics were 1.772, 1.837, and 1.898 for the psychological resilience, occupational stress, and emotional exhaustion models, respectively. Cook’s distance values were below 1 in all models. Overall, the assumptions of the regression analyses were considered to be met at an acceptable level.

Statistical significance was set at p < 0.05. Data analyses were performed using IBM SPSS Statistics for Windows, Version 27.0 (IBM Corp., Armonk, NY, USA) [37] and R version 4.3.1 (R Foundation for Statistical Computing, Vienna, Austria) [33].

### 2.4. Ethics Committee

Ethics committee approval was obtained from Batman University Ethics Committee to conduct the research (number: 2025/11-04; date: 13 November 2025). In addition, permissions for the use of all scales used in the research were obtained from the relevant researchers. Participants were informed about the purpose of the research, and their informed consent was obtained on a voluntary basis. Ethical principles were followed throughout the research process.

## 3. Results

According to Table 1, most participants are between 26 and 35 years old, with a similar ratio of women to men. The majority of participants hold a bachelor’s degree and are married. Regarding income, the most frequent report is that income is less than expenses. In terms of years of work experience, the majority have 5–10 years. Furthermore, a significant number of participants have been working shifts for 5 years or more.

According to Table 2, the measurement instruments demonstrated acceptable to high internal consistency. For the Workplace Violence Safety Perception Scale, Cronbach’s alpha and McDonald’s omega coefficients were 0.701 and 0.732 for the Safety subdimension and 0.901 and 0.921 for the Violence Management subdimension, respectively.

Male nurses reported lower occupational stress than female nurses (15.61 ± 3.76 vs. 16.82 ± 3.22; t = −3.524, *p* < 0.001, Hedges’ g = −0.346), higher psychological resilience (18.51 ± 3.82 vs. 17.46 ± 4.47; t = 2.563, *p* = 0.011, Hedges’ g = 0.251), lower emotional exhaustion (20.51 ± 6.49 vs. 21.78 ± 5.94; t = −2.092, *p* = 0.037, Hedges’ g = −0.204), and higher workplace violence safety perception (37.86 ± 12.23 vs. 34.28 ± 12.78; t = 2.929, *p* = 0.004, Hedges’ g = 0.286). No significant differences were observed across categories of shift-work duration for occupational stress (F = 1.456, *p* = 0.226, ω^2^ = 0.003), psychological resilience (F = 0.415, *p* = 0.743, ω^2^ < 0.001), emotional exhaustion (F = 1.466, *p* = 0.223, ω^2^ = 0.003), or workplace violence safety perception (F = 0.817, *p* = 0.485, ω^2^ < 0.001). As none of the ANOVA results were significant, post-hoc comparisons were not conducted.

### 3.1. Correlations Between Measurement Scores

**Figure 1:**
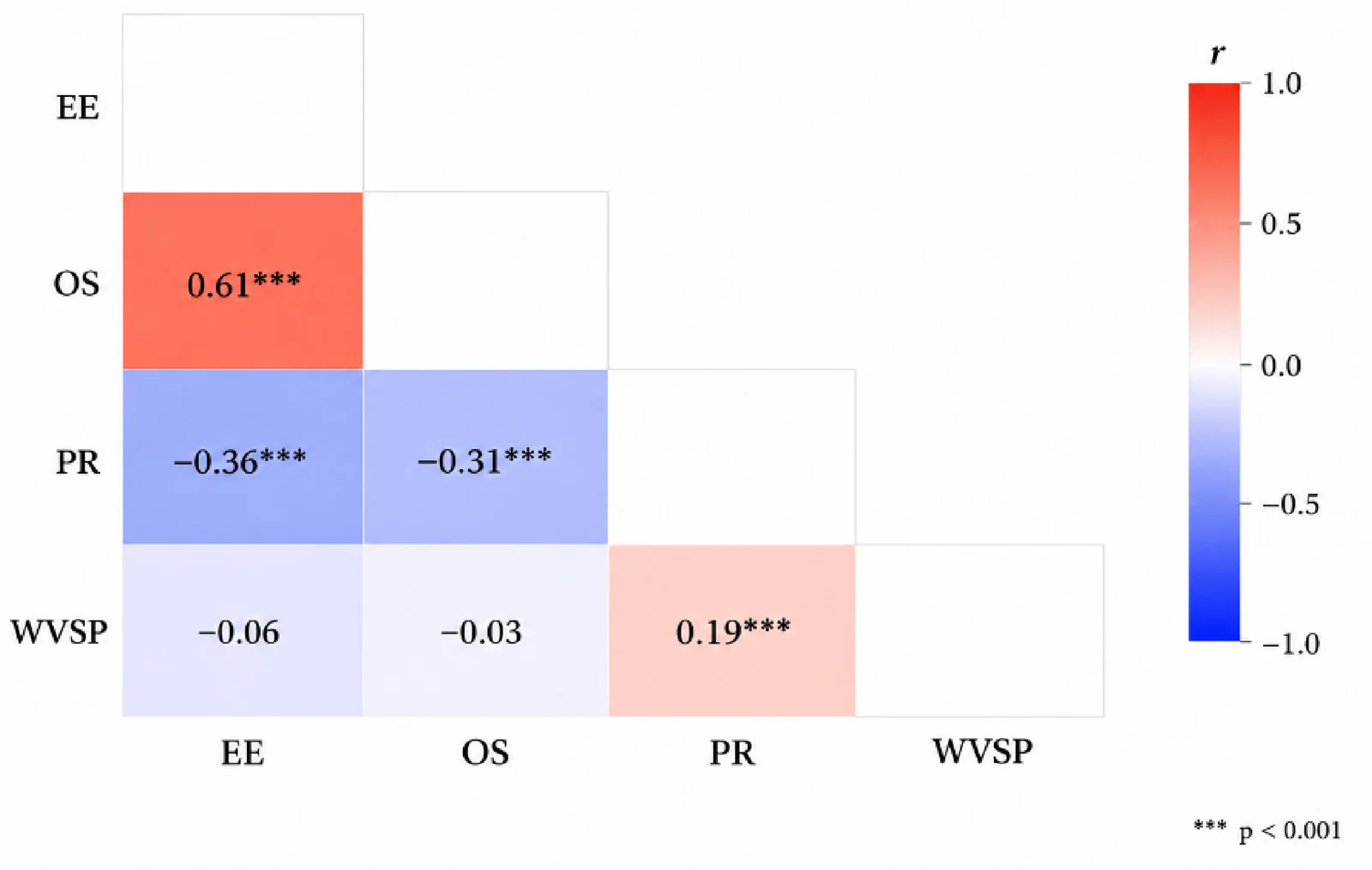
Correlation matrix of the study variables. Note. OS: Occupational Stress; PR: Psychological Resilience; EE: Emotional Exhaustion; WVSP: Workplace Violence Safety Perception.

According to Figure 1, higher occupational stress was associated with higher emotional exhaustion. Conversely, higher psychological resilience was associated with lower occupational stress and emotional exhaustion. A weak positive relationship was observed between psychological resilience and workplace violence safety perception. No significant relationships were found between workplace violence safety perception and either occupational stress or emotional exhaustion.

### 3.2. Indirect Association Model Including Psychological Resilience Between Workplace Violence Safety Perception and Emotional Exhaustion

After adjustment for sex, age group, years of work experience, and duration of shift work, workplace violence safety perception was positively associated with psychological resilience (B = 0.058, β = 0.174, *p* < 0.001). Higher psychological resilience was, in turn, associated with lower emotional exhaustion (B = −0.497, β = −0.334, *p* < 0.001). The direct association between workplace violence safety perception and emotional exhaustion was not statistically significant (B = 0.012, β = 0.025, *p* = 0.592). However, a significant indirect association through psychological resilience was observed (B = −0.029, β = −0.058, 95% bootstrap CI [−0.048, −0.013]). Given the cross-sectional design, this finding should be interpreted as a statistical indirect association rather than evidence of causal mediation.

**Figure 2:**
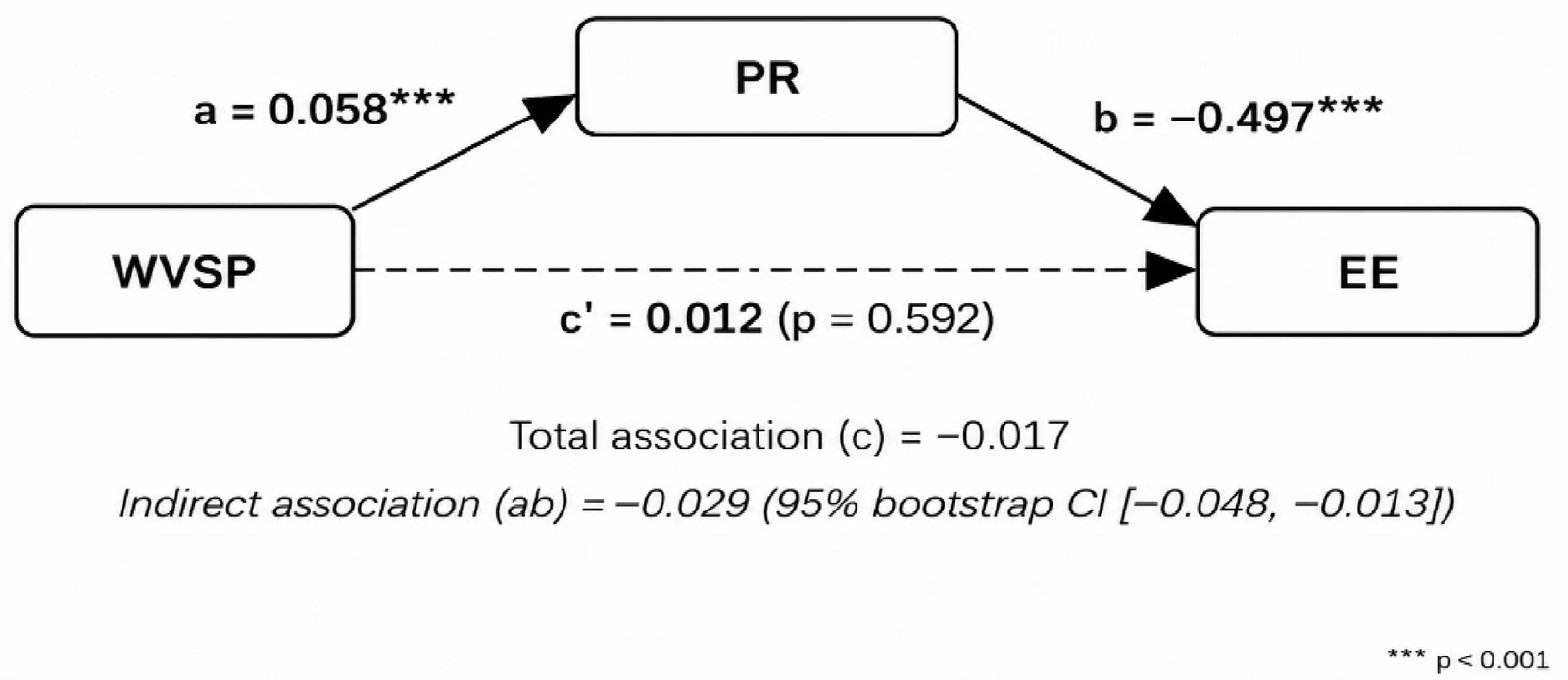
Indirect association model including psychological resilience between workplace violence safety perception and emotional exhaustion. Note. WVSP = workplace violence safety perception; PR = psychological resilience; EE = emotional exhaustion; CI = confidence interval.

Figure 2 presents the indirect association model including psychological resilience between workplace violence safety perception and emotional exhaustion. Arrows indicate the direction of the modeled associations.

According to Table 3, the perception of trust and safety against violence in healthcare was not directly and significantly associated with emotional exhaustion. However, this perception was positively associated with psychological resilience; higher psychological resilience was associated with lower emotional exhaustion. The significant indirect association, together with the non-significant direct association, suggests that psychological resilience may be involved in the relationship between workplace violence safety perception and emotional exhaustion. However, given the cross-sectional design, these findings should be interpreted as statistical indirect associations rather than evidence of causal mediation.

### 3.3. Indirect Association Model Including Psychological Resilience Between Workplace Violence Safety Perception and Occupational Stress

After adjustment for sex, age group, years of work experience, and duration of shift work, workplace violence safety perception was positively associated with psychological resilience (B = 0.058, β = 0.174, *p* < 0.001). In turn, higher psychological resilience was associated with lower occupational stress (B = −0.238, β = −0.283, *p* < 0.001). The direct association between workplace violence safety perception and occupational stress was not statistically significant (B = 0.013, β = 0.045, *p* = 0.329). However, a significant indirect association through psychological resilience was observed (B = −0.014, β = −0.049, 95% bootstrap CI [−0.023, −0.006]). Given the cross-sectional design, this result should be interpreted as a statistical indirect association rather than evidence of causal mediation.

**Figure 3:**
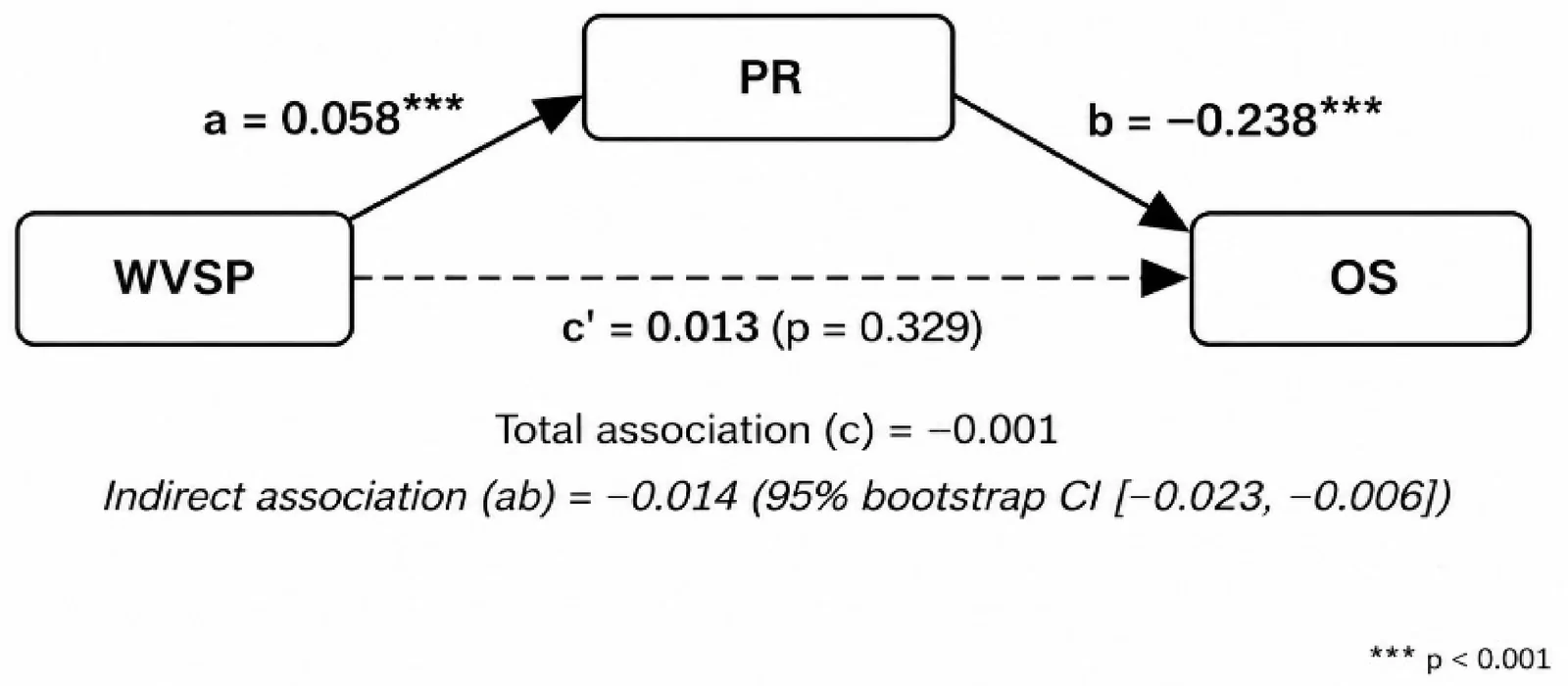
Indirect association model including psychological resilience between workplace violence safety perception and occupational stress. Note. WVSP = workplace violence safety perception; PR = psychological resilience; OS = occupational stress; CI = confidence interval.

Figure 3 presents the indirect association model including psychological resilience between workplace violence safety perception and occupational stress. Arrows indicate the direction of the modeled associations.

According to Table 4, perceptions of trust and security against violence in healthcare were not directly and significantly associated with occupational stress. However, perceptions of trust and security were positively associated with psychological resilience, and higher psychological resilience was associated with lower occupational stress. The significant indirect association, together with the non-significant direct association, suggests that psychological resilience may be involved in the relationship between workplace violence safety perception and occupational stress. However, given the cross-sectional design, these findings should be interpreted as statistical indirect associations rather than evidence of causal mediation.

According to Table 5, some of the hypotheses were supported. Higher perceptions of trust and safety against violence in healthcare were associated with higher psychological resilience, and higher psychological resilience was associated with lower occupational stress and emotional exhaustion. However, the perception of safety was not directly and significantly associated with occupational stress or emotional exhaustion. Psychological resilience was involved in significant indirect associations between workplace violence safety perception and both occupational stress and emotional exhaustion.

## 4. Discussion

This study aimed to examine the relationships among nurses’ perceptions of trust and safety regarding violence in healthcare, occupational stress, emotional exhaustion, and psychological resilience. The study found significant relationships among the variables. A positive correlation was found between occupational stress and emotional exhaustion, while psychological resilience was negatively correlated with occupational stress and emotional exhaustion. A weak positive correlation was found between psychological resilience and perceptions of trust and safety. However, no direct significant relationship was found between perceptions of trust and safety and occupational stress or emotional exhaustion.

When the hypotheses tested in the study were evaluated, hypothesis H1, which predicted a positive relationship between nurses’ perceptions of trust and safety against violence in healthcare and psychological resilience, was supported. This finding is consistent with studies indicating that safe and supportive work environments are associated with nurses’ psychological resilience [19,38,39]. Hypotheses H2 and H3, which predicted a negative relationship between psychological resilience and occupational stress and emotional exhaustion, were also supported. The literature reports that psychological resilience can be a protective resource for nurses against stress, traumatic experiences, and burnout [18,20,40]. In contrast, hypotheses H4 and H5, which predicted a direct negative relationship between perceptions of trust and safety against violence in healthcare and occupational stress and emotional exhaustion, were not supported. This finding suggests that occupational stress and emotional exhaustion may be associated with multiple factors beyond perceptions of safety. Indeed, factors such as workload, role conflict, time pressure, emotional demands, organizational support, and individual resources have been reported to influence stress and burnout [41,42,43,44,45]. Hypotheses H6 and H7 were also supported, indicating significant indirect associations among perceptions of trust and safety against violence in healthcare, psychological resilience, occupational stress, and emotional exhaustion. These findings suggest that perceptions of trust and safety may be indirectly associated with occupational stress and emotional exhaustion in models including psychological resilience. However, given the cross-sectional design of the study, these findings should be interpreted as statistical indirect associations rather than evidence of causal mediation. In this respect, the findings are consistent with studies showing that psychological resilience can be an important protective resource for nurses in the context of stress and burnout [19,20,46]. The study found a positive correlation between occupational stress and emotional exhaustion. Higher stress levels among nurses were associated with higher emotional exhaustion. This finding is consistent with both the national and international literature [42,43,47]. The relationship between occupational stress and burnout is particularly evident in professions requiring intense emotional labor, such as nursing. The literature indicates that factors such as workload and time pressure, complex and intense patient profiles, communication and emotional labor requirements, staff shortages, and lack of organizational support contribute to occupational stress [40,41,43]. High workloads, role conflicts, shift work, communicating with patients and their families, responding to crisis situations, and working under high responsibility may contribute to the accumulation and persistence of stress over time. This process may contribute to emotional exhaustion. Therefore, in work environments where there is a risk of violence in healthcare, occupational stress should be addressed not only as an individual problem but also within the scope of employee safety and psychosocial occupational health.

The literature indicates that psychological resilience is related to stress levels and is an important psychological resource supporting job satisfaction and mental well-being [48,49]. Studies have reported that psychological resilience can play a protective role against work stress and burnout among nurses [18,20,50]. A study conducted among nurses reported that workplace violence and burnout were associated with poorer psychological well-being. In addition, psychological resilience was found to weaken the negative association between workplace violence and psychological well-being [51]. In the present study, psychological resilience was negatively associated with occupational stress and emotional exhaustion. These findings suggest that nurses with higher levels of psychological resilience tend to report lower levels of occupational stress and emotional exhaustion. It has been reported that nurses with higher psychological resilience are better able to maintain their functioning despite challenging experiences such as psychological violence [49]. Furthermore, psychological resilience is influenced not only by individual characteristics but also by workplace conditions [19]. However, psychological resilience should not be considered solely an individual characteristic but should also be viewed in the context of workplace safety, organizational support, and occupational health practices.

The study found a positive correlation between nurses’ perception of safety and psychological resilience. Nurses feeling safer may be associated with a greater sense of control over their work environment and lower risk perception. Furthermore, employees who have not experienced violence have been found to have higher perceptions of safety [52]. The international literature shows that a strong sense of safety may be associated with higher employees’ resilience and that the organizational safety climate is positively related to psychological resilience [38,39]. A study conducted among emergency nurses reported that workplace violence was associated with psychological resilience and perceived organizational support and that both individual and organizational resources were important in work environments where there is a risk of violence [53]. Studies have shown that high patient volume, long waiting times, patient and family expectations, and lack of institutional support can weaken the perception of safety [54,55]. It is stated that the perception of safety should be considered not only in terms of physical safety measures but also in conjunction with elements such as organizational support, the functioning of reporting processes, and safety culture [52,56]. The findings of this study suggest that perceived safety may be associated with psychological resilience, which in turn is related to occupational stress and emotional exhaustion. The perception of a safe work environment may be associated with how nurses interpret emotional strain, perceive threats, and develop coping strategies. Therefore, a safe work environment is not only a risk mitigation factor but may also be associated with higher psychological resilience.

The study found significant indirect associations among workplace violence safety perception, psychological resilience, occupational stress, and emotional exhaustion. No significant direct association was observed between perceived safety and occupational stress or emotional exhaustion. However, indirect associations were observed in models including psychological resilience. This result suggests that psychological resilience may be considered not only an independent protective resource but also an important factor in relation to perceived safety and psychosocial outcomes. In the literature, psychological resilience has been identified as a protective factor in stress and burnout processes [19,46]. A study of intensive care nurses reported that those with higher levels of psychological resilience experienced lower levels of burnout despite traumatic experiences [18]. It has also been suggested that psychological resilience may help reduce the negative effects of emotional labor among nurses [20]. These findings suggest that in healthcare settings where there is a risk of violence, a sense of security alone may not be sufficient and that supporting psychological resilience is crucial for the psychosocial well-being of nurses.

The results of this research offer important implications for strengthening the professional continuity and sustainability of nurses. Policies regarding employee safety in healthcare institutions should not be limited solely to physical safety measures but should also include individual and institutional practices that support employees’ psychological resilience. In resource-limited settings, practical strategies may include brief in-service training, team debriefings following incidents of violence, and clear reporting procedures. Peer support and referral options for psychosocial assistance may also be considered for nurses who need additional support. It is stated that a safe working environment and supportive organizational conditions can strengthen nurses’ well-being at work [38]. Healthcare institution managers can contribute to increasing the psychological resilience of nurses by creating a safe working environment in institutions, emphasizing in-service training for nurses, strengthening collaboration and teamwork, and providing a strong communication network [19,39]. Furthermore, providing psychosocial support services to nurses and offering training in stress management, coping strategies, breathing exercises, and time management may help nurses manage occupational stress [20,40]. Furthermore, measures aimed at reducing excessive workloads may help nurses manage stress related to workload [44,45]. In this context, safety policies aimed at preventing violence in healthcare, effective incident reporting systems, and supportive organizational practices should be considered together in terms of both employee safety and psychosocial occupational health [2,54,55].

In conclusion, this study revealed the relationships between nurses’ perceptions of trust and safety against violence in healthcare, psychological resilience, occupational stress, and emotional exhaustion. The research findings showed a positive correlation between occupational stress and emotional exhaustion and a negative correlation between psychological resilience and occupational stress and emotional exhaustion. While perceptions of trust and safety against violence in healthcare did not show a direct significant relationship with occupational stress and emotional exhaustion, significant indirect associations were observed in models including psychological resilience. These findings suggest that nurses’ perceptions of a safe working environment should be considered together with their psychological resilience. It is recommended that practices aimed at strengthening employee safety in healthcare institutions should not be limited solely to physical safety measures; they should also include psychosocial occupational health practices that support psychological resilience, reduce occupational stress, and prevent emotional exhaustion.

## 5. Limitations

Due to the cross-sectional design of the study, the findings should be interpreted as associations rather than evidence of causal relationships or temporal ordering.

As all study variables were assessed using self-report measures within the same survey, the findings may also be subject to common-method bias. The observed associations may have been influenced by factors not measured in this study, including actual exposure to workplace violence, staffing levels, workload, unit characteristics, and organizational support. Hospital- and clinical unit-level differences were not examined in the adjusted models and may represent additional sources of unmeasured variation. Moreover, the workplace violence measure assessed nurses’ perceptions of safety and trust in violence management rather than actual exposure to workplace violence. The findings should therefore not be interpreted as reflecting the effects of direct exposure to workplace violence. The use of convenience sampling and data collection from only two public hospitals may limit the generalizability of the findings. In addition, using both online and face-to-face methods may have influenced participants’ responses.

## Figures and Tables

**Table 1:** Sociodemographic characteristics of the participants.

Variable	Category	n (%)
**Age**	Under 25	18 (4.3%)
	26–35	276 (65.9%)
	36–45	98 (23.4%)
	46–55	25 (6.0%)
	>55 years	2 (0.5%)
**Gender**	Male	203 (48.4%)
	Female	216 (51.6%)
**Educational Status**	High school	26 (6.2%)
	Bachelor’s degree	362 (86.4%)
	Master’s/Doctorate	31 (7.4%)
**Marital Status**	Single	154 (36.8%)
	Married	265 (63.2%)
**Income Status**	Income less than expenses	178 (42.5%)
	Income greater than expenses	92 (22.0%)
	Income equal to expenses	149 (35.6%)
**Years of Work Experience**	1–5 years	75 (17.9%)
	6–10 years	186 (44.4%)
	11–15 years	86 (20.5%)
	16–20 years	45 (10.7%)
	More than 20 years	27 (6.4%)
**Duration of Shift Work**	Less than 1 year	25 (6.0%)
	1–2 years	19 (4.5%)
	3–4 years	60 (14.3%)
	5 years or more	315 (75.2%)

n: number of observations.

**Table 2:** Descriptive statistics and internal consistency coefficients of the study variables (n = 419).

Variable	Mean ± SD	Min–Max	Median	α	ω
**Occupational stress**	16.23 ± 3.54	4–20	17	0.855	0.895
**Psychological resilience**	17.97 ± 4.20	6–30	18	0.768	0.801
**Emotional exhaustion**	21.17 ± 6.24	6–30	21	0.931	0.949
**Workplace violence safety perception**	36.01 ± 12.63	7–70	37	0.742	0.767
**Safety**	13.20 ± 7.36	3–30	13	0.701	0.732
**Violence management**	22.82 ± 9.37	4–40	23	0.901	0.921

SD: standard deviation; Min: Minimum; Max: Maximum; α: Cronbach’s alpha reliability coefficient; ω: McDonald’s omega reliability coefficient.

**Table 3:** Indirect association analysis including psychological resilience between workplace violence safety perception and emotional exhaustion.

Type	Association	B	SE	95% CI		β	t	*p*
				Lower Limit	Upper Limit			
**Indirect association**	Workplace violence safety perception ⇒ Psychological resilience ⇒ Emotional exhaustion	−0.029	0.009	−0.048	−0.013	−0.058	-	-
**Components**	Workplace violence safety perception ⇒ Psychological resilience	0.058	0.016	0.026	0.090	0.174	3.571	*p* < 0.001
	Psychological resilience ⇒ Emotional exhaustion	−0.497	0.068	−0.632	−0.363	−0.334	−7.279	*p* < 0.001
**Direct association**	Workplace violence safety perception ⇒ Emotional exhaustion	0.012	0.023	−0.032	0.057	0.025	0.537	0.592
**Total association**	Workplace violence safety perception ⇒ Emotional exhaustion	−0.017	0.024	−0.063	0.029	−0.034	−0.701	0.484

B: estimated regression coefficient, SE: standard error, CI: confidence interval, β: standardized regression coefficient, t: *t*-test statistic, *p*: significance level.

**Table 4:** Indirect association analysis including psychological resilience between workplace violence safety perception and occupational stress.

Type	Association	B	SE	95% CI		β	t	*p*
				Lower Limit	Upper Limit			
**Indirect association**	Workplace violence safety perception ⇒ Psychological resilience ⇒ Occupational stress	−0.014	0.004	−0.023	−0.006	−0.049	-	-
**Components**	Workplace violence safety perception⇒ Psychological resilience	0.058	0.016	0.026	0.090	0.174	3.571	<0.001
	Psychological resilience ⇒ Occupational stress	−0.238	0.039	−0.315	−0.162	−0.283	−6.110	<0.001
**Direct association**	Workplace violence safety perception⇒ Occupational stress	0.013	0.013	−0.013	0.038	0.045	0.978	0.329
**Total association**	Workplace violence safety perception ⇒ Occupational stress	−0.001	0.013	−0.028	0.025	−0.004	−0.086	0.931

B: estimated regression coefficient, SE: standard error, CI: confidence interval, β: standardized regression coefficient, t: *t*-test statistic, *p*: significance level.

**Table 5:** Hypotheses tested in the study and their support status according to the findings.

Hypothesis	Support Status
H1: Nurses’ level of trust and safety regarding violence in healthcare is positively correlated with their psychological resilience.	Supported. (r = 0.19, *p* < 0.05; also X → M path coefficient B = 0.058, *p* < 0.001)
H2: Psychological resilience level is negatively correlated with occupational stress level.	Supported. (r = −0.31, *p* < 0.05; also M → Y_1_ association B = −0.238, *p* < 0.001)
H3: Psychological resilience level is negatively correlated with emotional exhaustion.	Supported. (r = −0.36, *p* < 0.05; M → Y_2_ association B = −0.497, *p* < 0.001)
H4: Workplace violence safety perception is negatively associated with occupational stress.	Not supported. (OS–WVSP correlation: nonsignificant, *p* > 0.05; direct association B = 0.013, *p* = 0.329)
H5: Workplace violence safety perception is negatively associated with emotional exhaustion.	Not supported. (EE–WVSP correlation: nonsignificant, *p* > 0.05; direct association B = 0.012, *p* = 0.592)
H6: A statistically significant indirect association involving psychological resilience was observed between workplace violence safety perception and occupational stress.	Supported (significant indirect association; **B = −0.014, 95% bootstrap CI [−0.023, −0.006**]; direct association nonsignificant).
H7: A statistically significant indirect association involving psychological resilience was observed between workplace violence safety perception and emotional exhaustion.	Supported (significant indirect association; **B = −0.029, 95% bootstrap CI [−0.048, −0.013]**; direct association nonsignificant).

## Data Availability

Data related to the study are available from the corresponding author upon reasonable request.
